# Consumers of insect-based foods: a cross-cultural study between Belgium and Gabon

**DOI:** 10.1093/jisesa/ieae051

**Published:** 2024-05-04

**Authors:** Loïc Detilleux, Sandrine Bayendi Loudit, Philippe Le Gall, Frédéric Francis, Rudy Caparros Megido, Thomas Dogot

**Affiliations:** Economics and Rural Development, Gembloux Agro-Bio Tech—University of Liège, 5030 Gembloux, Belgium; Agricultural and Forestry Research Institute (IRAF), National Center for Scientific and Technological Research (CENAREST), Libreville, Gabon; UMR EGCE (Evolution, Genomes, Comportement, Ecologie), CNRS IRD—Paris-Sud University, 91190 Gif-sur-Yvette, France; Functional and Evolutionary Entomology, Gembloux Agro-Bio Tech—University of Liège, 5030 Gembloux, Belgium; Functional and Evolutionary Entomology, Gembloux Agro-Bio Tech—University of Liège, 5030 Gembloux, Belgium; Economics and Rural Development, Gembloux Agro-Bio Tech—University of Liège, 5030 Gembloux, Belgium

**Keywords:** entomophagy, novel food, European country, African country, willingness-to-pay

## Abstract

Human consumption of insects has previously been examined in cross-cultural studies. However, such studies rarely include African countries and willingness-to-pay for insect-based food has never been assessed in cross-cultural studies. The current study presents a cross-cultural study conducted with 409 urban dwellers from Belgium (191 males; 218 females) and 412 urban dwellers from Gabon (219 males; 193 females). Each respondent was surveyed with a questionnaire following the Knowledge, Attitude, and Practices model and included questions relative to willingness-to-pay for 2 insect-based foods (insect baguette and insect burger). More than 90% of respondents from both countries were familiar with edible insects. However, acceptance of entomophagy was lower in respondents from Gabon than in respondents from Belgium. Intercultural differences were also recorded between Gabonese ethnic groups. Most respondents who accepted entomophagy were willing to eat the insect baguette and/or the insect burger. These findings confirm that entomophagy could further develop in Belgium and Gabon. Willingness-to-pay varied between countries and between insect-based foods. In Belgium, the average prices of comparable conventional foods (i.e., same foods but without insects) were lower than the average willingness-to-pay for insect-based foods. In Gabon, respondents were not willing to pay extra for insect-based foods. Setting the right price for insect-based foods is a necessary step to promote more frequent insect consumption.

## Introduction

In 1975, Meyer-Rochow suggested that the World Health Organization (WHO) and the Food and Agriculture Organization (FAO) look at edible insects as a possible solution to the problem of global protein shortages (Meyer-Rochow 1975). Almost 40 years later, the FAO followed his recommendation and published a paper (“Edible insects: future prospects for food and feed security”) reporting on all aspects of insects as food and feed (e.g., nutrition, animal farming, history, environment, food safety, legislation, economics, etc.) for the first time. This report provided a benchmark for the insect value chain and boosted interest from both the public and private sectors. The trend was also followed by the scientific community with an increasing number of scientific articles, including cross-cultural studies relative to edible insects (van Huis et al. 2013, Mancini et al. 2022).

With globalization, the food industry tends to standardize food throughout the world while the perception of food generally differs from one culture to another (Ghosh et al. 2018, Jeong and Lee 2021). Such differences are highlighted by cross-cultural studies (Tan et al. 2015). Consequently, cross-cultural studies are essential for understanding the specific characteristics of each culture so as to develop appropriate food strategies. Most cross-cultural studies relating to entomophagy include European and/or Asian countries while cross-cultural studies including African countries are uncommon (Lensvelt and Steenbekkers 2014, Hartmann et al. 2015, Ruby et al. 2015, Tan et al. 2015). The few cross-cultural studies involving an African country included Ethiopia, Mozambique, or South Africa (Cunha et al. 2015, Castro and Chambers 2019a, 2019b, Ghosh et al. 2020).

The current cross-cultural study examines entomophagy in a European (i.e., Belgium) and an African country (i.e., Gabon). Belgian people, as most Westerners, generally do not eat insects, although some companies in Belgium produce insect-based foods (e.g., Aldento, Bugood Food, Kriket, etc.) (Van Thielen et al. 2018). However, Belgium was a European pioneer in approving 10 insect species for human consumption in 2014 (FASFC and SHC 2014). The list was implemented pending European legislation which appeared some years later with the marketing authorization for 4 insect species (Vale-Hagan et al. 2023). Many studies related to edible insects were conducted in Belgium to specifically identify factors supporting entomophagy development in Belgium and in other Western countries (Caparros Megido et al. 2014, 2016, Schouteten et al. 2016, Van Thielen et al. 2018, Detilleux, Wittock, et al. 2021). Three cross-cultural studies that included Belgium were also carried out; however, they were all based on the same online survey (Sogari et al. 2023, Tzompa-Sosa, Moruzzo, et al. 2023, Tzompa-Sosa, Sogari, et al. 2023). In Gabon, as in some other African countries (e.g., Democratic Republic of the Congo, Kenya, Nigeria, etc.), it is common for people to eat insects. A total of 75 species of edible insects are eaten in Gabon and entomophagy depends on culture, as some Gabonese ethnic groups consume more insects than other. However, to date, there has been only 1 study on entomophagy in Gabon (Detilleux, Poligui, et al. 2021).

Prior to becoming regular insect consumers, people must be willing to taste edible insects. The initial motivation for eating insects is often related to curiosity or desire to be more environmentally friendly (House 2016). Initial motivation has been assessed by several studies that included tasting sessions with edible insects (Caparros Megido et al. 2014, 2016, Lensvelt and Steenbekkers 2014, Schouteten et al. 2016, Sogari et al. 2017, Tan et al. 2017, García-Segovia et al. 2020, Petersen et al. 2020). Integrating insects into daily menus appears to be influenced by more practical factors such as taste, availability, compatibility with current habits, and price (House 2016, Ghosh et al. 2018). In the field of marketing, pricing is a complex strategy that requires knowledge about potential buyers, including their willingness-to-pay (WTP). WTP is defined as “the highest price an individual is willing to accept to pay for some good or service,” and it is influenced by the perceived value and the utility of the good/service (Breidert 2005). Different methods exist to estimate WTP, and they are based on market data, experiments, or surveys (Breidert et al. 2006). Many studies on entomophagy have included a measurement of WTP for insect-based foods. Most of the time, insect-based foods studied were familiar foods (e.g., pasta, buns, cookies, sushi, etc.) containing non-visible insects (Pascucci and de-Magistris 2013, Alemu et al. 2017, Collins et al. 2019, Lombardi et al. 2019, Giotis and Drichoutis 2021). Such features are recommended to make insect-based foods more readily accepted (Caparros Megido et al. 2016).

The aim of the current cross-cultural study was to explore the potential of developing insect-based foods in Belgium and Gabon. Such aim focused on consumers and was divided into 3 secondary objectives: (i) to characterize and compare entomophagy in Belgium and Gabon, (ii) to examine ethnic disparities in Gabon regarding entomophagy, and (iii) to assess the WTP for 2 insect-based foods. These secondary objectives were performed using a Knowledge, Attitude, and Practices questionnaire. This type of questionnaire distinguishes between what the participants know about entomophagy (i.e., Knowledge), what they are willing to do (i.e., Attitude), and what they actually do (i.e., Practices) (Gumucio et al. 2011). Such questionnaires are used to record Knowledge, Attitude, and Practices around specific topics (e.g., entomophagy) and to target future strategies (e.g., promotion of edible insects). For example, the assessment of knowledge helps to develop information and education programs by identifying areas where efforts are still required (Gumucio et al. 2011).

## Materials and Methods

### Study Design

Data were collected from surveys with respondents from Belgium and Gabon. Both countries are characterized by predominantly urban populations whose purchasing habits are oriented toward supermarkets and the consumption of processed foods (Central Intelligence Agency (CIA) 2023a, 2023b, The Global Alliance for Improved Nutrition (GAIN) 2023). Such features were particularly relevant to the current study. Respondents were recruited from 2 cities in each country: Namur (N50°27’59”–E4°51’58”) and Ottignies Louvain-la-Neuve (N50°39’55”–E4°33’37”) in Belgium; and Libreville (N0°24’31”-E9°26’31”) and Franceville (S1°38’4”–E13°35’22”) in Gabon. Namur and Ottignies Louvain-la-Neuve are located in Wallonia (i.e., the French-speaking part of Belgium), and they are one of the most populated urban areas in their respective province (Statbel 2022). Libreville is the capital of Gabon and the largest city of the country, while Franceville is located further south-east and is the main city of the Haut-Ogooué Province (Direction Générale de la Statistique 2015, Central Intelligence Agency (CIA) 2023a).

Surveys were conducted at the entrance to supermarkets, with permission from the managers. Each customer was invited to participate for a few minutes in an anonymous survey related to scientific research. Once agreed, customers answered a questionnaire that was read and recorded by a surveyor (i.e. 1 of the authors of the current study). The survey was conducted in French with data collected on a tablet in Belgium and on paper in Gabon. The questionnaire was designed with Typeform (Barcelona, Spain) on the tablet and with Microsoft Word v.2016 (Santa Rosa, California, USA) on paper. The days and times of data collection and the supermarkets were varied to obtain a variety of respondent profiles. On average, the administration of a questionnaire took 1 min and 40 s per respondent. However, the length of the questionnaire (from 9 to 16 questions) depended on the participants’ answers (see below). Surveys were conducted over a 2-month period in 2022: throughout April in Gabon and from mid-June to mid-July in Belgium. The cross-cultural study received ethical approval (no. 20211224) from the Human and Social Sciences Ethics Committee of University of Liège.

Sociodemographic parameters of each participant including gender, age, nationality, native language (only for Gabonese people), education level, and monthly income level were collected from participant declarations. Concerning questions relative to education and income levels, the list of possible answers varied between Belgium and Gabon to reflect the reality of the country. However, the answer modalities of each country were grouped into categories for comparisons between countries. The native languages of Gabonese people were grouped into language groups based on the work of Mouguiama-Daouda (2005).

After the surveys, data were excluded from participants who were younger than 18 as no parental consent was obtained. In addition, data from non-European respondents in Belgium and non-African respondents in Gabon were excluded to limit the cultural complexity of the study. In all, 19 surveys were excluded, leaving a total of 409 respondents in Belgium and 412 respondents in Gabon. In Belgium, 56.23% of the data came from Ottignies Louvain-la-Neuve, while 57.28% of the Gabonese data were collected in Libreville. The sociodemographic profile of the respondents from both countries is presented in Table 1.

**Table 1.** Sociodemographic profile of respondents—overall and by country

		Total (*n* = 821)	Belgium (*n* = 409)	Gabon (*n* = 412)
Gender	Female	411	218	193
	Male	410	191	219
Age class	[18–25]	148	101	47
	[26–40]	309	98	211
	[41–65]	315	165	150
	>65	49	45	4
Nationality	Native	754	381	373
	Non-native	67	28	39
Education level	None	12	3	9
	Very low	18	5	13
	Low	296	113	183
	Medium	217	137	80
	High	278	151	127
Monthly income level	No answer	75	31	44
	Very low	168	85	83
	Low	244	133	111
	Medium	224	124	100
	High	110	36	74
Native language	Fang	–	–	99
	Mbete	–	–	59
	Ndjabi	–	–	39
	Punu	–	–	81
	Teke	–	–	45
	Other	–	–	50

Nationality: native (Belgian; Gabonese), non-native (another nationality than Belgian; Gabonese). Education level; answers were grouped into 5 categories: none, very low (primary school for both), low (secondary school for Belgium; secondary school and baccalaureate degree for Gabon), medium (bachelor’s degree for Belgium; licence degree for Gabon), and high (master’s degree and higher for both). Monthly income level; answers were grouped into 5 categories: no answer, very low (< 1,000 € for Belgium; < 80,000 FCFA for Gabon), low (1,000–2,000 € for Belgium; 80,000–250,000 FCFA for Gabon), medium (2,000–3,000 € for Belgium; 250,000–600,000 FCFA for Gabon), and high (> 3,000 € for Belgium; > 600,000 FCFA for Gabon); €: euro; FCFA: Franc of the Financial Community of Africa. Native language (for natives from Gabon only); answers were grouped into 6 language groups based on the work of Mouguiama-Daouda (2005): Fang, Mbete, Ndjabi, Punu, Teke, and Other (Other = several language groups with low number of representatives in the study).

### Knowledge, Attitude, and Practices Relative to Entomophagy

The first question focused on Knowledge, and introduced the topic of the study: “Do you know that some insects can be eaten by humans?” (entomophagy knowledge). Then, participants were questioned about their knowledge of edible insect supply points (supply knowledge): “Do you know where to get edible insects?” and “If yes, where?” with one or several answers ranging from markets, specialist shops, supermarkets, restaurants, webshops, in nature, and other.

Attitude of respondents was investigated through acceptance of entomophagy (“Would you accept to eat insects?”). Respondents who refused to eat insects were asked to specify the reason(s) for refusal from several statements: “insects are scary,” “eating insects is dangerous,” “I get no benefit,” “I do not need it,” “It is not in my culture,” “I am allergic to insects,” “I do not want to taste new food,” “Eating insects is disgusting,” “I do not have enough information about this food,” and “other.”

Questions relating to Practices were only answered by respondents who accepted eating insects (entomophagy acceptors). Such participants were categorized into insect eaters and insect non-eaters with the following question: “Have you already consumed edible insects?” (entomophagy experience). Insect eaters specified their consumption frequency of edible insects by selecting the most appropriate answer: several times/week, once/week, several times/month, once/month, several times/year, or once/year (frequency).

### WTP for Insect-Based Foods

Before the WTP assessment, entomophagy acceptors had to choose which insect-based food they would eat (none, one, or both) from a baguette made of insect flour (i.e., insect baguette) and an insect burger. Their choice was based on names and pictures of foods. Pictures were images of the comparable conventional foods (i.e., same foods but without insects) to highlight that insect-based foods were similar by showing no sign of visible insects, as previously recommended (Caparros Megido et al. 2016). Then, respondents received the payment card(s) corresponding to their food choice. The payment card is a WTP assessment method that has recently been used in studies on entomophagy (Giotis and Drichoutis 2021, Sogari et al. 2022). In this method, participants were placed in a hypothetical purchasing scenario, and they were asked to select the amount that most closely matched the maximum amount they were willing to pay for a food item (Riccioli et al. 2020). Payment cards were country- and food-specific: the median amount was the average price of similar conventional food and other amounts corresponded to plus and minus 10%, 25%, 50%, and 100% of this average price. In the questionnaire, the amount “minus 100%” was replaced by the statement “No desire to purchase.” The average price for each food was determined during an exploratory survey in several outlets of each country. After completing the payment card(s), participants had to select the expected benefit(s) of the selected food(s) compared to conventional food(s). Their choice was made from a list of several types of benefits: environmental, nutritional, sensorial, economical, ethical, other, or none.

### Statistical Analysis

All statistical tests were performed with Minitab v.19 (State College, PA, USA) for Windows. Chi-square tests were applied on the Knowledge, Attitude, and Practices data to identify similarities and differences between countries and between Gabonese ethnic groups. Regarding WTP for insect-based foods, a 1-sample *t*-test was applied in each country to compare WTP to the average of the comparable conventional food. Several tests used ΔWTP instead of WTP because the price difference between the 2 insect-based foods and the currency difference between the 2 countries made the WTP comparison irrelevant. ΔWTP represented the relative difference between WTP for insect-based food and the average price of comparable conventional food. The first test on ΔWTP involved an analysis of variance (ANOVA) with a generalized linear model (GLM) for all sociodemographic factors, except nationality which was correlated with other factors: 2 (country) × 2 (gender) × 4 (age class) × 5 (education level) × 5 (monthly income level). The purpose of this test was to determine whether the homeland (i.e., factor “Country”) had an influence on ΔWTP for insect-based foods and whether this influence was due to any interaction with another sociodemographic factor. Then, statistical differences found by the GLM were tested with a Kruskal–Wallis test, as the assumptions of 1-way ANOVA (i.e., population normality and variance homogeneity) were not met. Finally, multiple comparisons were evaluated using Dunn’s test. These 2 tests enabled a more in-depth study of the previously identified influences. The same approach was applied to examine the difference of ΔWTP between the 2 insect-based foods.

## Results

### Knowledge, Attitude, and Practices Relative to Entomophagy

Entomophagy knowledge was more widespread in Belgium than in Gabon (chi-square = 23.423, *P* < 0.001). Despite this statistical difference, over 90% of respondents in each country were acquainted with this food practice (98.78% in Belgium and 91.50% in Gabon). A reverse trend was observed for supply knowledge (chi-square = 43.818, *P* < 0.001), as the percentage of respondents who identified edible insect supply points was higher in Gabon (66.50%) than in Belgium (43.52%). Nature was the most frequently cited supply point in Gabon (64.60%), followed by markets (54.01%), and restaurants (10.58%). In Belgium, the most frequent answer was specialist shops (87.08%), followed by webshops (38.20%), and supermarkets (25.28%).

**Fig. 1.**
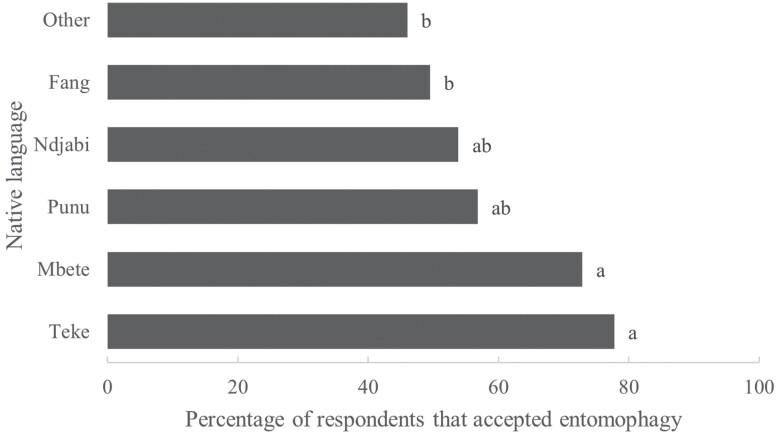
Percentage of entomophagy acceptors in relation to their native language (*n* = 373). Different superscript letters indicate a significant difference.

Regarding attitude, urban people from Belgium were more prone to eat insects than people from Gabon (chi-square = 10.495, *P* = 0.001; acceptance in Belgium: 68.22%; acceptance in Gabon: 57.28%). Reasons given for refusal to eat insects were similar in both countries, with “It is not in my culture” (33.08% in Belgium and 46.02% in Gabon) and “Eating insects is disgusting” (39.23% in Belgium and 42.61% in Gabon) as the most frequent responses. In Gabon, acceptance of entomophagy varied between ethnic groups (chi-square = 18.827, *P* = 0.002; Fig. 1).

**Fig. 2.**
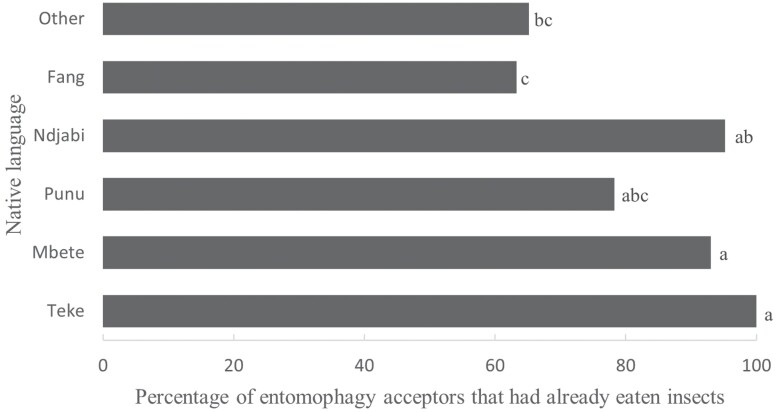
Percentage of insect eaters in Gabon by native language. The percentages were calculated based on entomophagy acceptors (*n* = 217). Different superscript letters indicate a significant difference.

Among entomophagy acceptors, there were more respondents from Gabon who ate insects (81.78%) than respondents from Belgium (67.74%; chi-square = 13.152, *P* < 0.001). In Gabon, the ethnic group of the respondents influenced entomophagy experience (chi-square = 29.613, *P* < 0.001; Fig. 2) with the most experience in the Teke ethnic group. In both countries, most insect eaters (93.12% in Belgium and 58.02% in Gabon) consumed edible insects once/year.

### WTP for Insect-Based Foods

**Fig. 3.**
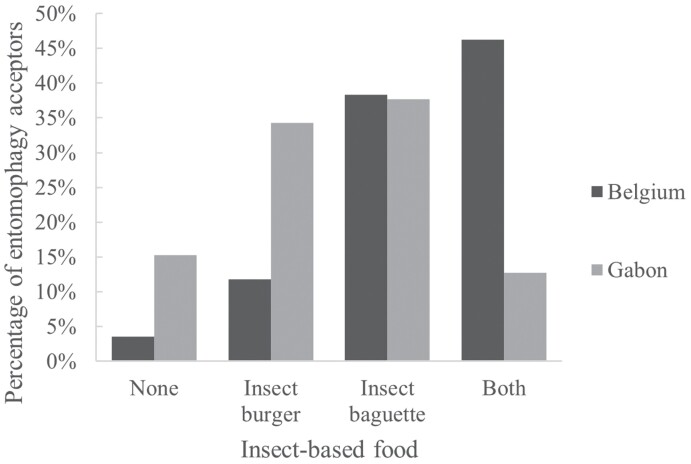
Choice of insect-based food. Percentages were calculated based on entomophagy acceptors (nBelgium = 279 and nGabon = 236).

A total of 269 participants from Belgium responded that they would eat at least 1 of the insect-based foods, with a strong preference for the insect baguette, as 84.59% of entomophagy acceptors said they would eat this food. In Gabon, 84.75% of entomophagy acceptors were ready to eat the insect baguette and/or the insect burger (Fig. 3).

The GLM for the WTP for an insect burger found an interaction between the factors “Country” and “Education level” (Table 2). However, the Kruskal–Wallis test did not confirm this effect, as there was no significant difference between education levels within the same country (Belgium: *H*_4,162_ = 5.78, *P* = 0.216; Gabon: *H*_4,111_ = 6.41, *P* = 0.171). Findings relative to the correlation between education level and WTP for the insect burger were similar. Education level was identified as a discriminatory factor by the GLM (Table 2), but was not significant according to the Kruskal–Wallis test (*H*_4,273_ = 6.66, *P* = 0.155).

**Table 2.** Results of the GLM on the ΔWTP (%) for the insect-based foods

Factor	Insect baguette			Insect burger		
	DF	*F*	*P*	DF	*F*	*P*
Country	1	7.62	**0.006**	1	35.94	**<0.001**
Gender	1	3.38	0.067	1	0.05	0.828
Age class	3	0.36	0.783	3	0.66	0.580
Education level	4	1.21	0.307	4	3.54	**0.008**
Monthly income level	4	1.08	0.366	4	1.01	0.401
Country * Education level	–	–	–	4	2.56	**0.039**

ΔWTP for insect baguette and insect burger: relative difference between WTP for insect-based food and the average price of the comparable conventional food. Country: Belgium and Gabon. Gender: male and female. Age class: [18–25], [26–40], [41–65], and > 65 years old. Education level: none, very low, low, medium, and high. Monthly income level: no answer, very low, low, medium, and high. DF: degree of freedom. *F*: F-statistic. *P*: significance level. Bold data indicate significant results. Only significant interaction effects are included in the table, but all others were checked when it was possible (i.e., when interaction was not correlated with other factors/interactions of the GLM or when interaction had enough different combinations of the interacting factors).

The influence of the homeland (i.e., factor “Country”) on the WTP for insect-based foods was also observed (Table 2). The ΔWTP for both insect-based foods was greater in participants from Belgium than respondents from Gabon (Table 3).

**Table 3.** ΔWTP (%) for the insect-based foods (mean value ± standard deviation)

	Belgium	Gabon	Statistical analysis	*P*
Insect baguette	15.34 ± 36.53^a^	3.66 ± 34.70^b^	*H*_1,355_ = 10.37	**0.001**
Insect burger	8.36 ± 27.25^a^	−13.74 ± 34.66^b^	*H*_1,273_ = 37.60	**<0.001**

ΔWTP for insect baguette and insect burger: relative difference between WTP for insect-based food and the average price of comparable conventional food. Different superscript letters indicate significant differences between countries. Bold data indicate significant results.

**Fig. 4.**
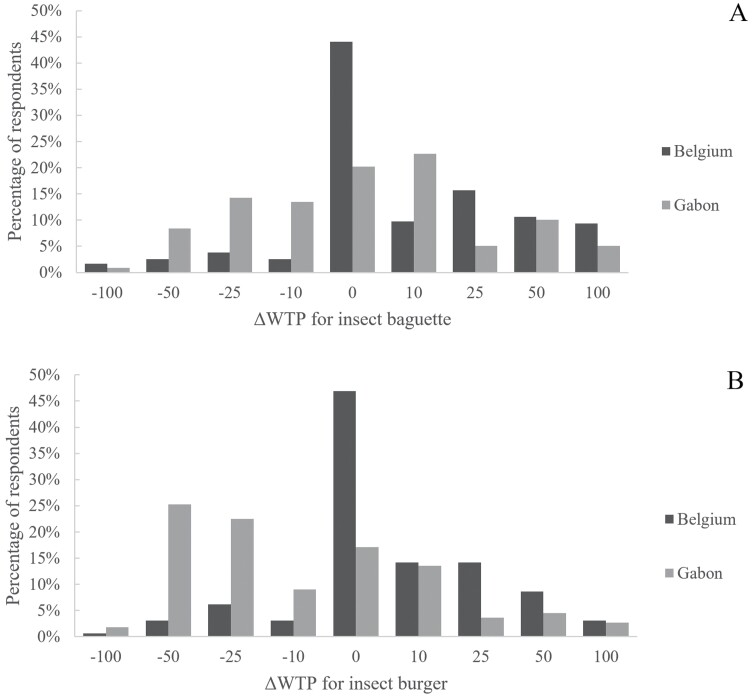
Distribution of ΔWTP (%) for A) insect baguette and B) insect burger. The ΔWTP was calculated by the relative difference between WTP for insect-based food and the average price of the comparable conventional food. The percentages of respondents were calculated from those that were willing to eat the insect-based food (insect baguette: nBelgium = 236 and nGabon = 119; insect burger: nBelgium = 162 and nGabon = 111).

Approximately 50% of participants from Belgium would pay the same price for an insect-based food compared to a similar conventional food (Fig. 4). In Belgium, on average, respondents were willing to pay extra for an insect baguette (*T* = 6.45, *P* < 0.001) and an insect burger (*T* = 3.91, *P* < 0.001). In Gabon, the average WTP for the insect baguette was not significantly different than the average price of a conventional baguette (*T* = 1.15, *P* = 0.253). However, the WTP for an insect burger was lower than a conventional burger for respondents from Gabon (*T* = −4.18, *P* < 0.001).

The ΔWTP was higher for the insect baguette (ΔWTP = 11.42 ± 36.30) than for the insect burger (ΔWTP = −0.62 ± 32.31; *H*_1,628_ = 16.13, *P* < 0.001).

In both Belgium (64.31%) and Gabon (78.50%), the most frequently noted benefit associated with entomophagy was nutrition. In contrast, 20.07% of respondents from Belgium and 10.50% of respondents from Gabon cited no benefit from eating insect-based foods.

## Discussion

### Knowledge, Attitude, and Practices Relative to Entomophagy

Entomophagy knowledge and acceptance of eating insects were higher in respondents from Belgium than in respondents from Gabon, whereas entomophagy experience was lower in Belgium than in Gabon. The dynamics of entomophagy were significantly different between Belgium and Gabon.

In Belgium, entomophagy is viewed as a new food habit advertised by the media, scientists, and some companies (Van Thielen et al. 2018, Detilleux, Wittock, et al. 2021). Eating insects is mainly promoted as a healthy and sustainable food practice that contributes to food well-being among Western consumers (Batat and Peter 2020). This message may be important to some individuals and could spark curiosity to try insect-based food, which is a key predictor of consumer acceptance (Sogari et al. 2017, Stone et al. 2022). The growing awareness led to an increase in knowledge about entomophagy in Belgium over the last few years: from 61.9% in 2013 to 78.7% in 2017 and 98.8% in 2022 (Caparros Megido et al. 2014, Van Thielen et al. 2018). However, the European entomophagy sector is therefore still in the development phase: the first species have only recently been approved for marketing, the start-ups are becoming well-established companies, the investments are increasing, and insect-based foods are diversifying (Payne et al. 2019, Mancini et al. 2022, Vale-Hagan et al. 2023). In this context of developing novelty, insect-based foods are still scarce in stores. In the current study, respondents from Belgium thought that edible insects were mainly sold in specialist shops. This knowledge likely corresponds to the current market situation in Belgium. Participants also claimed to act like traditional Westerners, as their consumption events were scarce and likely limited to special events and travel (Tan et al. 2015, Detilleux, Wittock, et al. 2021).

Concerning Gabon, as in other cultures familiar with entomophagy, urban dwellers tend to modernize their diet and avoid traditional eating habits, such as consuming edible insects (Vantomme 2015, Müller 2019). The lower Knowledge, Attitude, and Practices related to entomophagy might therefore be explained by the high level of urbanization in Gabon. Moreover, as recorded in another Gabonese study, attitude and practices toward edible insects were different between ethnic groups with the Fang and the Punu as the least entomophagic groups (Detilleux, Poligui, et al. 2021). The Fang and the Punu were precisely the largest ethnic groups in Gabon, but also in the current study (Central Intelligence Agency (CIA) 2023a). Belonging to an ethnic group is the best predictor of food preferences and influences the selection of insect species and the way they are cooked (Ghosh et al. 2018). In the current study, supply knowledge was consistent with the history and existing market of entomophagy in the country. In many African countries, edible insects have been a habitual food item for many years in a subsistence economy. In most of these countries, insects are collected in the wild for household consumption or bought in the consumer market. The Gabonese consumer markets are also supplied by imports of edible insects from neighboring countries (Muafor et al. 2015, Ebenebe et al. 2020). Regarding practices, most participants with entomophagy experience reported consuming edible insects only once per year. Such finding is in agreement with a prior study in Gabon, suggesting that edible insects are considered a delicacy and are eaten only on special occasions or possibly by the seasonal availability of edible insects (Bomolo et al. 2017, Detilleux, Poligui, et al. 2021).

Despite the differences between Belgium and Gabon, reasons for rejection of insect-eating were similar in both countries with disgust and cultural incompatibility as the most cited reasons. This finding is consistent with prior literature (Ruby et al. 2015, Sogari et al. 2017, Van Thielen et al. 2018, Kröger et al. 2022).

### WTP for Insect-Based Foods

Only 8.9% of all entomophagy acceptors said they would refuse to eat an insect baguette and/or an insect burger. Such foods are salty and show no obvious visual signs of insects, meeting expectations of potential insect-based food consumers relative to flavor and appearance (Tan et al. 2015, 2017, Caparros Megido et al. 2016, Detilleux, Wittock, et al. 2021). However, respondents were unable to consider certain parameters that may have influenced their decision, such as the odor of the foods (Ghosh et al. 2018). These foods did not exist in the Belgian or Gabonese market but similar conventional foods were familiar in both countries. The present study was the first to use insect baguette; however, similar bakery foods with insects such as bread, biscuits, cookies, brownies, etc. were previously proposed in several tasting sessions (Lensvelt and Steenbekkers 2014, Alemu et al. 2017, Sogari et al. 2017, García-Segovia et al. 2020, Petersen et al. 2020). The insect burger has already been used in studies evaluating insect-based foods, as insects are often seen as meat substitute (Caparros Megido et al. 2016, Schouteten et al. 2016).

The WTP for an insect baguette and/or an insect burger was not influenced by sociodemographic factors, except by culture. Prior literature on WTP for insect-based food has also reported no effect of gender and education level (Collins et al. 2019, Lombardi et al. 2019, Giotis and Drichoutis 2021). The results of the current study, reporting that income does not influence WTP for insect-based food, are in agreement with Collins et al. (2019); but they are contrasted with Giotis et al. (2021) which reported that income positively affects WTP (Collins et al. 2019, Giotis and Drichoutis 2021). While the current study found no influence of age on WTP for insect-based food, this correlation varies greatly across the literature. Two studies report that age and WTP are negatively associated, while another study reports a positive association (Collins et al. 2019, Lombardi et al. 2019, Giotis and Drichoutis 2021). Variability in the findings pertaining to age and acceptance of insect-based food have also previously been reported (Kröger et al. 2022). Concerning the influence of culture, respondents from Belgium had a higher WTP for both insect-based foods than respondents from Gabon. Further research is needed to study the influence of sociodemographic factors on WTP, especially for culture, as this study is the first cross-cultural study that assessed WTP for insect-based foods.

The current study found that WTP varied by product, as respondents from both countries had a higher WTP for the insect baguette than for the insect burger. These findings are in agreement with Lombardi et al. (2019) who stated that carriers (i.e., baguettes and burgers in this study) influenced WTP for insect-based foods (Lombardi et al. 2019). In Belgium, respondents were on average willing to pay extra for the insect baguette and the insect burger. This trend is in agreement with Alemu et al. (2017), but it differs from 2 other studies finding that most people would pay less for insect-based foods compared to similar conventional foods (Collins et al. 2019, Giotis and Drichoutis 2021). The difference in findings may be explained by the carrier used. The current study used the baguette and the burger, the study of Alemu et al. (2017) used bread, whereas other studies have used minced meat, energy bars, and cookies. Compared with these 3 foods, the baguette, the burger, and the bread are more familiar, ready to eat, and salty. These attributes are known factors in favoring insect consumption (Caparros Megido et al. 2014, 2016, Lensvelt and Steenbekkers 2014, Hartmann et al. 2015, Tan et al. 2015, 2017, Collins et al. 2019, Detilleux, Wittock, et al. 2021). The visual aspect of insect baguettes and insect burgers (i.e., no visible sign of insect) is also likely a key factor, as visible insects in food tend to negatively affect WTP for insect-based foods (Pascucci and de-Magistris 2013). Other studies have reported that individuals were willing to pay more for insect-based foods or for poultry fed with insects when they were first informed of the benefits of entomophagy (Lombardi et al. 2019, Sogari et al. 2022). In the current study, respondents were not informed about edible insects, however, the majority were already aware of the benefits associated with insect consumption. Similar to other studies, nutrition was the most frequently cited benefit among respondents (Ruby et al. 2015, Van Thielen et al. 2018). As suggested by those respondents, edible insects are nutritious. However, the nutritional profile of insects varies greatly between species and is quite similar to that of meat. Promoting the insect burger over a conventional meat burger for its nutritional value is therefore inadequate, especially with certain insect species that are rich in energy, sodium, and saturated fats, which can aggravate problems related to overnutrition (Payne et al. 2016, Orkusz 2021). This statement is particularly true for Belgium and Gabon, where obesity rates are increasing. However, in Gabon, an increasing proportion of the population is also undernourished (FAO 2021). For the latter, consuming food enriched with edible insects, such as insect baguettes, can be an effective way to combat nutritional deficiencies (Payne et al. 2016).

Despite the average WTP being higher for insect-based foods, many respondents from Belgium wanted to pay the same price both for foods with and without insects. These results suggest that insect-based foods should not be excessively higher priced than conventional foods, as this will discourage insect consumption (Tuccillo et al. 2020). Such situation is currently applied in European countries and represents a barrier to the development of entomophagy (House 2016). In contrast, the WTP for an insect burger was lower for respondents from Gabon. An explanation for this finding could be that the average price of a conventional burger, used as a reference, was perceived as too high. By judging a product combination (i.e., here, insects in burger), respondents also judged the appropriateness of the combination. In the case of unfamiliar food, food appropriateness has more impact than some relevant factors as sensory liking and an inappropriate combination could lead to a low WTP (Tan et al. 2016, Lombardi et al. 2019).

## Limitations of This Study

The contingent valuation method used to measure the WTP for insect-based foods has several flaws: respondents tend to overlook the foods’ characteristics and the constraints associated with buying foods (e.g., availability of foods or their competitors, household budget, etc.) to focus only on prices of foods; their estimations of WTP remain statements and can differ from their real attitude; even if their attitude reflects reality, it may never translate into practices; estimating WTP for unfamiliar foods as insect-based foods is difficult for respondents; etc. (Breidert et al. 2006, Le Gall-Ely 2009). In the case of this study, the foods were only presented as pictures, and they were not present in the markets of both countries. WTP for insect burger and insect baguette were therefore evaluated on the basis of respondents’ prejudices (e.g., level of interest, expected benefits, neophilia, etc.) rather than their experience with these foods (Caparros Megido et al. 2016).

In Gabon, edible insects are more readily available during the rainy season (from September to December), whereas the Gabonese data for this study were collected in April. Therefore, it is possible that this seasonal pattern influenced the question about the consumption frequency of edible insects.

## Conclusions

Most urban dwellers from both Gabon and Belgium were accepting of including edible insects in their diet. Advertisement from the entomophagy sector seems effective in Belgium, as participants were aware and curious about eating insects. In Gabon, knowledge and acceptance of entomophagy were lower than that in Belgium but this may be partially due to the modernization of food habits which occurs especially in urban areas. The edible insect sector in Gabon could therefore draw on the Belgian advertising strategy to improve the acceptance of entomophagy. People from Gabon had more experience in eating insects than respondents from Belgium, but they both consumed them infrequently. Many respondents were acquainted with the benefits of entomophagy, especially nutritional benefits. In Gabon, ethnic groups differed in their acceptance and practice of insect-eating.

Many respondents who accepted the consumption of edible insects were willing to eat both the insect baguette and the insect burger. Such insect-based foods could potentially be successfully marketed if entomophagy develops. However, more studies on optimal formulation, sensory liking, etc. are necessary to develop a more favorable product. Other carriers are also promising, but they need to have characteristics such as being salty, familiar, or without obvious visual signs of insects. In Belgium, the average WTP for insect-based foods was higher than the average prices of comparable conventional foods while participants from urban Gabon were willing to pay less for an insect burger and the same price for an insect baguette compared to similar conventional foods. In both countries, it is crucial for the edible insect sector to refrain from setting excessively high prices to avoid hindering the growth of entomophagy. WTP varied between the 2 insect-based foods and between Belgium and Gabon. However, cross-cultural studies assessing WTP should be replicated to confirm the influence of culture on WTP. The impact of other sociodemographic factors, such as age or income, on WTP for insect-based food also warrants further attention.
